# The Impact of Pre- and Post-Treatment Processes on Corrosion Resistance of Micro-Arc Oxidation Coatings on Mg Alloys: A Systematic Review

**DOI:** 10.3390/ma18030723

**Published:** 2025-02-06

**Authors:** Jiuwei Chi, Hongliang Zhang, Shuyu Song, Weisheng Zhang, Xingyu He, Zhisheng Nong, Xue Cui, Teng Liu, Tiannan Man

**Affiliations:** School of Materials Science and Engineering, Shenyang Aerospace University, Shenyang 110136, China; abc15725723569@163.com (J.C.); 18304241006@163.com (S.S.); 15056932685@163.com (W.Z.); xingyuhe0413@163.com (X.H.); nzsfir@126.com (Z.N.); papercx@163.com (X.C.); tliu@sau.edu.cn (T.L.); manxiaoxiao165@163.com (T.M.)

**Keywords:** Mg alloys, corrosion resistance, micro-arc oxidation, pre-treatment, post-treatment

## Abstract

As one of the lightest metallic structural materials, magnesium (Mg) alloys possess numerous distinctive properties and are utilized across a broad spectrum of applications. However, the poor corrosion resistance of Mg alloys limits their application. Micro-arc oxidation (MAO) is an effective surface treatment method that enhances the corrosion resistance of Mg alloys. Nevertheless, the intrinsic porous structure of MAO coatings hinders significant improvement in corrosion resistance. Research indicates that the pre- and post-treatment processes associated with MAO markedly enhance the densification of the oxide coatings, thereby improving their overall performance. This paper aims to provide a comprehensive review and analysis of the effects of various pre- and post-treatment processes, highlighting key advancements and research gaps in improving MAO coatings on Mg alloys. An in-depth analysis of the crucial role of pre-treatment in optimizing interfacial bonding and post-treatment in enhancing coating density is conducted using electrochemical testing and scanning electron microscopy (SEM). Finally, the future development of pre- and post-treatment processes are discussed.

## 1. Introduction

**Figure 1:**
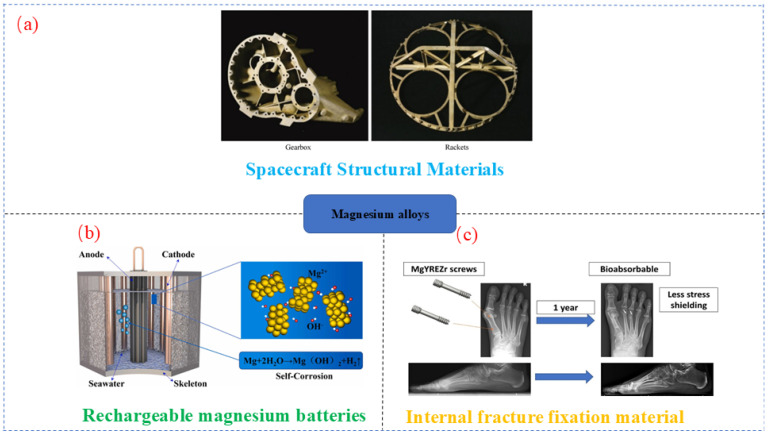
Application of Mg alloys in spacecraft (**a**) [28], battery technology (**b**) [29], and biomaterials (**c**) [30].

Magnesium (Mg) alloys have found extensive applications in the aerospace [1,2,3], automotive [4,5,6,7], electronics [8], and biomedical industries [9,10,11,12] owing to their lightweight nature [13,14], high specific strength, excellent thermal conductivity, effective shock absorption capabilities, superior electromagnetic radiation resistance, and biodegradability [15,16,17]. Figure 1 highlights the diverse applications of Mg alloys in the fields of spacecraft [18,19,20], battery technology [21,22,23], and biomaterials [24,25,26,27].

However, Mg is a highly reactive metal element with a standard electrode potential of (−2.37 V), which is more negative than that of commonly used metals such as iron (−0.45 V), titanium (−1.63 V), and aluminum (−1.66 V). Pure Mg readily forms a brittle and porous MgO coating in open air, while it tends to generate a protective Mg(OH)_2_ layer with low solubility in humid environments or electrolytes. Nevertheless, the facile spalling of the Mg(OH)_2_ layer leads to re-exposure of Mg to the corrosive environment, thereby accelerating the corrosion process. Mg alloys are susceptible to various forms of corrosion, typically involving galvanic corrosion between the second phase, such as the β-phase (Mg_17_Al_12_) in the Mg-Al alloy system [31,32], or impurities and the matrix. This results in the formation of unstable hydroxide films, which cause dissolution of the matrix. The inadequate corrosion resistance of Mg alloys limits their prospective applications [33,34,35]. At present, the principal techniques for improving the corrosion resistance of Mg alloys are alloying [36,37,38] and applying surface protective coatings [39,40].

**Figure 2:**
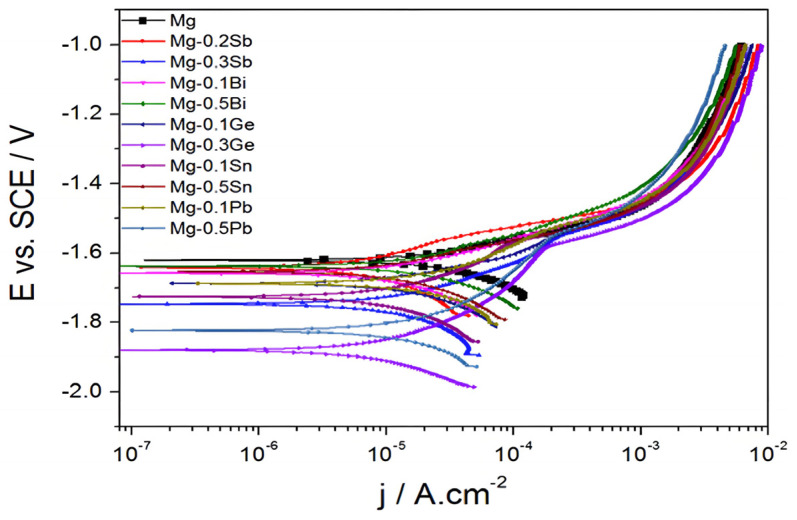
Potentiodynamic Polarisation Results of Pure Mg and Binary Mg Alloys in 0.1 M NaCl (pH = 6) [44].

The incorporation of elements such as Al [41], Mn [42], Zn [41,42], Ca [37,41,42], and Y [43] has been demonstrated to enhance the corrosion resistance of Mg alloys to varying degrees. This improvement can be attributed to two potential mechanisms. First, alloying elements can prevent cathode reaction or galvanic corrosion of Mg alloys, thereby enhancing the corrosion resistance of the matrix. Secondly, they can form a dense passivation coating on the surface of the matrix, which effectively protects Mg alloys from corrosion. Figure 2 illustrates the impact of varying concentrations of distinct alloying elements on the corrosion potential (*E_corr_*) and corrosion current density (*I*_corr_) of pure Mg. It is noteworthy that the Mg-0.2Sb alloy displays slightly enhanced corrosion resistance in comparison to pure Mg, whereas the Mg-0.3Sb alloy exhibits slightly diminished corrosion resistance. The findings of this study indicate that alloying can enhance the corrosion resistance of Mg alloys to a certain extent, emphasizing the importance of selecting the optimal alloying element content. Nevertheless, most alloying applications are aimed at enhancing the mechanical attributes of Mg alloys, with comparatively limited impact on corrosion resistance [37,38,44].

Preparing corrosion-resistant coatings on Mg alloys involves various methods, including conversion coating, sol-gel [45,46,47], electroless plating [48,49,50], electroplating [51], anodic oxidation [52], and micro-arc oxidation (MAO) [53,54,55]. Among these techniques, MAO has gained considerable attention due to its simplicity, high efficiency, and environmentally friendly nature, which makes it an attractive option for many applications. It is capable of producing composite oxide coatings with good adhesion and substantial thickness on valve metals, including aluminum, magnesium, and titanium [56,57,58]. MAO technology utilizes plasma generated by high-voltage discharges to enable the in-situ growth of thicker, protective coatings on the surface of Mg alloys. Table 1 presents the *E_corr_* and *I_corr_* of the MAO coatings prepared on Mg alloys. A composite ceramic coating with dispersed hydroxyapatite (HA) nanoparticles was prepared on the surface of AZ31 Mg alloy using MAO [59]. The study revealed that the *E_corr_* of the coating was −1.156 V, higher than that of the AZ31 alloy (−1.536 V). In a related study, Y. Vangölü et al. [53] developed a Zn-doped MAO coating on AZ31 Mg alloy, resulting in an increase of *E_corr_* value to −0.71 V.

**Figure 3:**
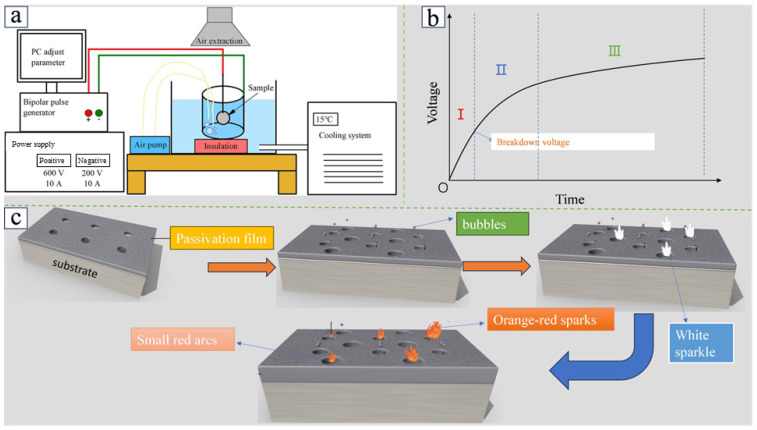
Schematic diagram of MAO equipment (**a**) [60]. Voltage curve of MAO process over time (**b**) [61,62]. Schematic diagram of the mechanism of MAO of Mg alloys (**c**).

Figure 3a illustrates the MAO apparatus, where the sample and a stainless-steel container serve as electrodes, powered by a dual-polarity pulse supply. The positive terminal of the power supply is connected to the sample, while the negative terminal is connected to the stainless-steel container. A refrigeration system is typically utilized to maintain a constant temperature for the electrolyte throughout the MAO process. The integration of a stirrer into the electrolyte enables the uniform distribution of the coating composition and ensures a constant temperature throughout the coating preparation process. Figure 3b depicts the MAO coating preparation process, wherein the voltage undergoes a series of changes over time. These changes can be broadly classified into three stages: (I) the formation of the passivation coating and the anodic oxidation stage, (II) the spark discharge stage, and (III) the MAO stage. When the voltage is below the breakdown voltage, the sample undergoes passivation and anodic oxidation, resulting in the formation of a thin insulating oxide coating. The insulating oxide coating is disrupted, resulting in the appearance of numerous minute white sparks on the material surface when the voltage exceeds the breakdown voltage, which is designated the “spark discharge stage”. As the voltage continues to increase, large and moving red arcs appear on the surface. Subsequently, the red arcs increase in size and depth, ultimately forming an orange-red spark. Figure 3c provides a schematic representation of the MAO coating preparation process for Mg alloys, delineating the fundamental stages: the passivation stage, anodic oxidation stage, spark discharge stage, and the MAO stage.

**Table 1:** Electrochemical parameters (*E_corr_* and *I_corr_*) of the MAO coatings formed on Mg alloys.

Substrate	Material Composition	Composition of the MAO Electrolyte	Electrical Parameters of the MAO Process	Substrate		MAO		Ref.
				*E_corr_* (V)	*I_corr_* (μA/cm^2^)	*E_corr_* (V)	*I_corr_* (μA/cm^2^)	
AZ31	Al 2.34 ωt.%, Mn 0.19 ωt.%, Zn 0.63 ωt.%, Fe 0.01 ωt.%, Mg Bal	12 g/L Na_3_PO_4_∙12H_2_O, 6 g/L NaOH	Voltage: 400 V, Duty cycle: 10%, Frequency: 100 Hz, Oxidation time: 600 s	−1.57	151	−1.51	0.017	[63]
	Mg 96%, Al 3%, Zn 1%	15 g/L Na_2_SiO_3_, 5 g/L KF	Voltage: 430 V, Oxidation time: 15 min	−1.48	3.77	−1.42	0.031	[64]
	Al 9.1 ωt.%, Zn 0.85 ωt.%, Mn 0.27 ωt.%, Fe ≤ 0.02 ωt.%, others ≤ 0.01 wt.%, Mg balance	10 g/L Na_2_SiO_3_, 4 g/L NaOH, 5 g/L NaF, 5 g/L (NaPO_3_)_6_	Voltage: 400 V, Frequency: 1000 Hz, Duty cycle: 10%, Oxidation time: 10 min	−1.21	26.72	−1.425	0.034	[65]
	Al 2.5–3.5 ωt%, Zn 0.7–1.3 ωt%, Mn 0.2–1.0 ωt%, Si 0.05 ωt%, Cu 0.01 ωt%, Fe ≤ 0.005 ωt%, Ni ≤ 0.005 ωt% and balanced Mg	0.04 M Na_2_SiO_3_·H_2_O, 0.1 M KOH, 0.2 M KF·2H_2_O	Current density: 50 mA·cm^−2^, Frequency: 300 Hz, Duty cycle: 10%, Oxidation time: 15 min	−1.51	14.02	−1.64	0.047	[66]
	-	12.5 g/L Na_2_SiO_3_, 5 g/L KOH	Voltage: 750 V, Current density: 0.1 A·cm^−2^, Duty cycle: 40%, Frequency: 100 Hz	-	-	−1.44	0.18	[67]
AZ31B	Ca 0.006 ωt%, Zn 0.96 ωt%, Si 0.01 ωt%, Ni 0.01 ωt%, Fe 0.006 ωt%, Mn 0.39 ωt%, Al 2.65 ωt% and Surplus Mg	3 g/L Na_3_PO_4_·12H_2_O, 3 g/L KOH, 2 g/L ZnO, 3 g/L HA	Current density: 300 mA·cm^−2^, Frequency: 1000 Hz, Duty cycle: 50%, Oxidation time: 7 min	−1.54	7.034	−1.156	0.034	[59]
AZ91D	Al 9.4 ωt%, Zn 0.82 ωt%, Mn 0.23 ωt%, Si 0.01 ωt%, Cu 0.02 ωt%, Ni 0.002 ωt%, Fe 0.005 ωt% and remainder Mg	25 g/L Na_2_SiO_3_·9H_2_O, 20 g/L KOH, 5 g/L KF·2H_2_O, 5 g/L C_6_H_5_Na_3_O_7_·2H_2_O, 5 g/L C_3_H_8_O_3_	Current density: 50 mA·cm^−2^, Duty cycle: 10%, Frequency: 300 Hz	−1.55	37	−1.391	0.053	[68]
ZK61		20 g/L (NaPO_3_)_6_, 3 g/L NaOH, 5 g/L EDTA-2Na, 5 g/L NaF	Current density: 5 A·dm^−2^, Frequency: 200 Hz Duty cycle: 15%, Oxidation time: 10 min	−1.53	112.1	−1.482	6.327	[69]

In silicate systems, micro-arc oxidation of magnesium alloys with diverse elements leads to the formation of various phases. For pure magnesium, MgO and Mg(OH)_2_ are prevalent. MgO, featuring a cubic crystal structure, offers high hardness and chemical stability, thus strengthening the film. Mg(OH)_2_, with its layered structure, fills film pores and improves corrosion resistance. When aluminum is present in the magnesium alloy, MgAl_2_O_4_ (spinel phase) and Al_2_O_3_ emerge. MgAl_2_O_4_, cubic in structure, has excellent thermal and chemical stability along with high hardness, enhancing the film’s wear and corrosion resistance. Al_2_O_3_ exists in different crystal forms like α-Al_2_O_3_, which is highly stable and hard, and γ-Al_2_O_3_, known for its large specific surface area and good adsorption. In zinc-containing magnesium alloys, ZnO and Zn(OH)_2_ are formed. ZnO, a semiconductor with a hexagonal structure, can alter the film’s electrical properties and has antibacterial features. Zn(OH)_2_ fills pores and has a relatively low decomposition temperature. Regarding silicon in the silicate system, it forms Mg_2_SiO_4_ (forsterite phase) and SiO_2_. Mg_2_SiO_4_, with an orthorhombic structure, improves film compactness and its corrosion and wear resistance. SiO_2_ boosts the film’s hardness and chemical stability. For calcium-containing magnesium alloys, CaSiO_3_ (wollastonite phase) and CaO are generated. CaSiO_3_, with a triclinic structure, enhances high-temperature and chemical corrosion resistance. CaO, an alkaline oxide, buffers acidic substances and increases film hardness. In magnesium alloys with manganese, MnO and Mn_2_O_3_ are formed. Both with cubic structures, MnO has chemical activity to regulate film composition and properties, while Mn_2_O_3_ improves the film’s oxidation resistance. These phases significantly influence the performance of magnesium alloy micro-arc oxidation films.

**Figure 4:**
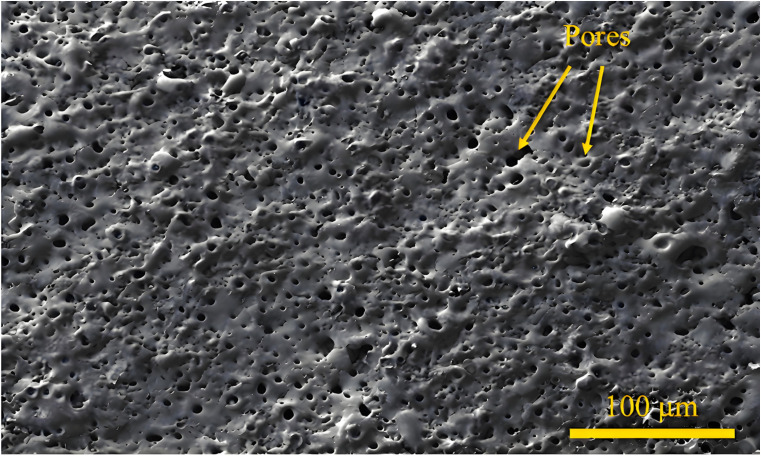
SEM images of the sample surface of a MAO-coated WE43 specimen [56].

It is evident that intrinsic defects, such as micro-pores and micro-cracks, are likely to form within the MAO coating based on the working principle of MAO technology. Figure 4 shows the typical porous structure of the MAO coating on Mg alloy, which is beneficial for the corrosive medium to penetrate into the Mg matrix [56]. The formation of micro-pores and micro-cracks can be attributed to a number of factors. First of all, the rapid solidification of molten oxides in the electrolyte can induce thermal stresses, thereby generating micro-cracks in the coating structure [70]. In addition, the continuous micro-arc discharge channels result in the formation of micro-pores on the coating surface. Ultimately, the primary constituent of the MAO coating, magnesium oxide (MgO), exhibits a Pilling–Bedworth Ratio (PBR) value of 0.81 < 1, which is another major reason for its porous structure [71]. The presence of micro-pores and micro-cracks within the coatings serves as a conduit for corrosive ions to diffuse through, thereby reducing the effectiveness of the barrier against corrosive chemical media and consequently reducing the corrosion resistance. In light of this understanding, researchers in both domestic and international contexts are inclined towards enhancing the corrosion resistance of the MAO coatings on Mg alloys by either pre- or post-treatment processes.

**Figure 5:**
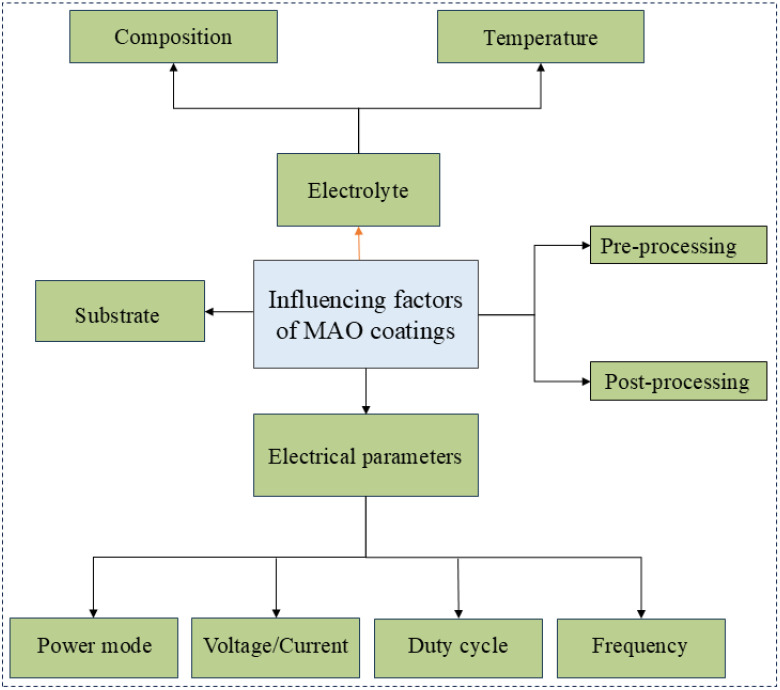
Factors influencing the properties of MAO coating on magnesium alloys.

A multitude of factors influence the performance of MAO coatings, including the substrate material, electrolyte, electrical parameters, additives, pre- and post-treatment processes illustrated in Figure 5. The composition and types of alloying elements present in different Mg alloys have the potential to influence the phase composition, microstructure, and properties of MAO coatings. For instance, the β-phase intermetallic compound present in the AZ91D alloy has been demonstrated to result in the formation of vertical pores, whereas the AZ31 alloy has been shown to give rise to spherical pores. Furthermore, the characteristics of the power supply operating mode can also exert an influence on the properties of the MAO coatings. Coatings formed under constant voltage mode exhibit the highest roughness, thickness, and uniform corrosion resistance, while films formed under constant power mode have the lowest surface roughness and possess the best pitting corrosion resistance [72,73]. The electrical parameters are of great consequence with regard to the corrosion resistance of the MAO coatings. For example, the application of a negative voltage in constant voltage mode has been demonstrated to increase the density of the coating, thereby enhancing its corrosion resistance. The termination voltage is found to be obviously correlated with the thickness and density of the coating [74]. As the terminal voltage increases, the roughness of the coating rises while its density diminishes. Increasing the frequency reduces the energy per breakdown event, which decreases the size of the discharge pores, increases coating density, and improves corrosion resistance [75,76]. The electrolyte exerts a profound direct influence on the MAO coating of Mg alloys. Among the most commonly utilized electrolytes, silicates and phosphates have been demonstrated to markedly enhance corrosion resistance. The conductivity and concentration of the electrolyte exert an influence on the arc initiation voltage, which in turn affects the size of the micro-pores in the coating, thereby impacting the structure and corrosion resistance of the coating. An electrolyte with an excessively high pH value can result in the dissolution of the MAO coating on Mg alloys, thereby reducing its corrosion resistance [77,78]. Pre-treatment of the substrate material before MAO enhances the corrosion resistance of MAO coatings by improving the interfacial bonding strength between the coating and the substrate, as well as reducing defects such as porosity and micro-cracks on the coating surface. Post-treatment improves the density of the MAO coating on Mg alloys by sealing pores or forming composite coatings, thereby further enhancing corrosion resistance.

During recent years, in addition to several studies examining other influencing factors [79,80,81]. there has been a surge in research on the impact of pre- and post-treatment processes on the corrosion resistance of MAO coatings on Mg alloys. Therefore, this paper systematically reviews the impact of pre- and post-treatment processes on corrosion resistance of MAO coatings on Mg alloys.

At present, the pre- and post-treatment processes have a significant impact on the structure and performance of the micro-arc oxidation coating of Mg alloys. However, no review on this aspect has been reported so far. This paper summarizes the influence laws of the pre- and post-treatment processes on the structure and performance of the MAO film by sorting out their working principles, technical characteristics and effects on the MAO process of magnesium alloys. This work provides detailed theoretical basis and technical support for relevant researchers, scholars, and engineers.

## 2. Effect of Pre-Treatment Process

The pre-treatment process phase entails the implementation of a targeted treatment regimen on the magnesium alloy substrate in advance of the MAO procedure. This is done with the goal of optimizing the surface microstructure and overall structure. The application of an appropriate pre-treatment process has the potential to enhance the interfacial bonding strength between the coating and substrate, as well as reduce surface porosity, micro-cracks, and other defects in the MAO coating, thereby improving its corrosion resistance. The most commonly employed pre-treatment process for Mg alloy before MAO include laser treatment [82,83,84,85,86], MS [87,88], and CS [89], as evidenced in the literature. Table 2 provides a summary of the ways in which these pre-treatment processes contribute to enhancing the corrosion resistance of MAO coatings.

**Figure 6:**
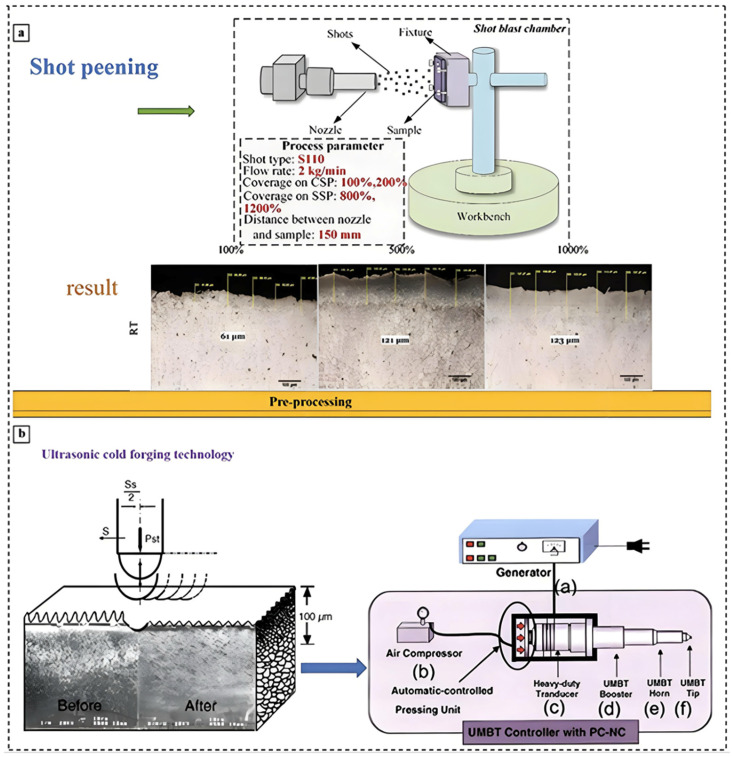
Equipment & Diagrams for Common Pre-treatment: SP Process & Results (**a**) [90,91]; MS Apparatus & Performance (**b**) [92].

**Table 2:** *E_corr_* and *I_corr_* of MAO Coatings on Mg Alloys with/without Pre-treatment.

Substrate	Pre-Treatment Process	MAO Coatings Without Pre-Treatment Process		MAO Coatings With Pre-Treatment Process		Ref.
		*E_corr_* (V)	*I_corr_* (A·cm^−2^)	*E_corr_* (V)	*I_corr_* (A·cm^−2^)	
AZ31	CS			−1.15	5.18 × 10^−7^	[89]
	SP	−1.611	7.11 × 10^−7^	−1.62	2.14 × 10^−7^	[93]
	MS			−1.46	1.552 × 10^−6^	[87]
				−1.25	3.93 × 10^−6^	[88]
	UCFT	−1.415	18.6 × 10^−6^	−1.31	9.20 × 10^−6^	[94]
	cerium conversion	−0.395	0.34 × 10^−6^	−0.25	1.18 × 10^−9^	[95]
AZ91D	LSP	−0.148	6.4 × 10^−8^	−1.47	5.00 × 10^−9^	[96]
				−1.39	4.22 × 10^−7^	[97]
Mg alloy	LSM			−1.49	8.24 × 10^−7^	[98]

Note. CS—Cold Spray; SP—Shot Peening; MS—Magnetron Sputtering; UCFT—Ultrasonic Cold Forging Technology; LSP—Laser Shock Processing; LSM—Laser Surface Melting; Cerium conversion is a surface treatment technology mainly used to improve the corrosion resistance of metals such as aluminum alloys, magnesium alloys, etc. The basic principle is to prevent the erosion of corrosive media by forming a layer of cerium-containing compounds (such as cerium hydroxide or cerium oxide) on the surface of the metal as a protective film.

As depicted in Figure 6, the pre-treatment stage encompasses a diverse array of methodologies, primarily laser treatment, magnetron sputtering (MS), and shot peening (SP) among others. These processes are primarily employed to enhance the adhesive strength of the subsequent coating, which is achieved by altering the microstructural characteristics of the Mg alloy surface. In the case of MS, a thin coating is deposited onto the Mg alloy surface, which serves as a foundational layer upon which the MAO coating is subsequently formed.

### 2.1. Surface Laser Process

Laser processing technology employs the use of high-power laser beams to facilitate the heating, melting, and evaporation of materials from the surface. This results in alterations to the grain size and chemical composition of the substrate surface layer due to the high thermal gradient and rapid cooling rates [99,100]. This technique has attracted considerable interest within the field of material preparation due to its high efficiency and precision. Laser treatment of Mg alloys not only results in the homogenization of the surface microstructure but also facilitates the dissolution of the second phase in the matrix [101], thereby creating favorable conditions for the preparation of MAO coatings with excellent comprehensive performance.

**Figure 7:**
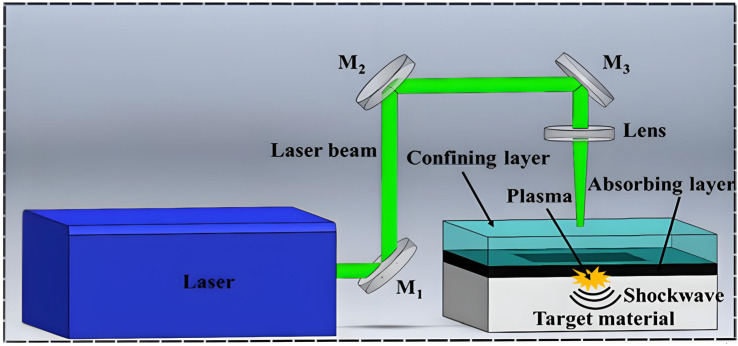
Schematic view of the LSP experimental setup [102].

Laser Shock Processing (LSP), also referred to as Laser Peening (LP), is a process that generates intense shockwaves on the surface through the utilization of high-power pulsed laser beams. This process has the potential to induce compressive residual stresses in the material [101], thereby enhancing the surface structure of Mg alloys and positively impacting the generation of MAO coatings [82,83]. As illustrated in Figure 7, the LSP process involves coating the sample surface with an absorbing layer and a limiting layer. Subsequently, the pulsed laser beam is focused on the absorbing layer, which absorbs the laser energy and generates a high-temperature and high-pressure plasma. The generation and subsequent expansion of nanosecond laser-generated plasma into the ambient gaseous medium, due to the presence of a limiting layer, results in the transmission of a shock wave to the target material. This phenomenon is caused by the outward diffusion of the plasma being constrained, which occurs as a consequence of the plasma being generated and expanding into the surrounding medium. When the shock pressure exceeds the elastic limit of the material, plastic deformation occurs, resulting in the creation of deep compressive residual stresses in the surface area. Defects such as twins and dislocations are formed as a consequence of the process. The aforementioned defective structures serve to enhance the stability of residual stress through the pinning effect. Moreover, the crystal structure of the material is refined. Furthermore, Xiong et al. [84] demonstrated that the combination of LSP and MAO markedly enhances the corrosion resistance of AZ80 Mg alloys. This enhancement is primarily attributed to the refinement of grain size and the formation of a nanocrystalline coating, which effectively reduces the adsorption of Cl^−^ on the surface and promotes the formation of a MgO passivation layer as a result of LSP pre-treatment. Upon penetration of the MAO coating by a simulated body fluid (SBF) solution, a dense passivation coating is immediately formed on damaged portions in the vicinity of the exposed surface of the nanocrystalline layer. The coating serves as a self-healing agent, facilitating the repair of damage caused by the SBF solution.

**Figure 8:**
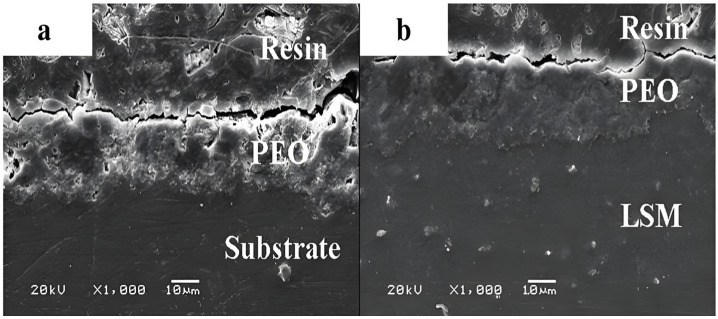
Cross-sectional SEM micrographs of MAO (**a**) and LSM–MAO (**b**) treated specimens [96].

In addition to LSP, the corrosion resistance of MAO coatings on Mg alloys can be further enhanced through laser surface melting (LSM). The LSM technique allows for precise control over the microstructure of the material, thereby optimizing the surface structure and grain size of Mg alloys [85,86]. The research conducted by Wang [96] and Liu [97] demonstrated that LSM pre-treatment distinctly enhanced the corrosion resistance of MAO coatings on AZ91D alloys. Figure 8 depicts the cross-sectional morphology of untreated and treated MAO coatings, revealing a huge reduction in micro-pores in the pretreated coatings. Furthermore, Chen et al. [103] achieved the successful production of a denser MAO coating on AZ91D alloy surfaces using LSM pre-treatment, resulting in a substantial improvement in corrosion resistance. The dissolution and redistribution of β-phase induced by LSM led to a reduction in its content, effectively mitigating galvanic coupling corrosion between α-Mg matrix and β-phase. This reduction in galvanic coupling corrosion prevents the formation of concentrated corrosion on large areas of α-Mg matrix, thus averting severe localized corrosion. The impact of laser treatment technology on the augmentation of corrosion resistance in MAO coatings is typically not considerable (*I_corr_* is reduced by about one order of magnitude). This is primarily due to the low elastic limit of Mg, which, even at low laser intensities, can result in intrinsic deformation of the material, potentially influencing the fatigue life of the material.

### 2.2. Magnetron Sputtering

**Figure 9:**
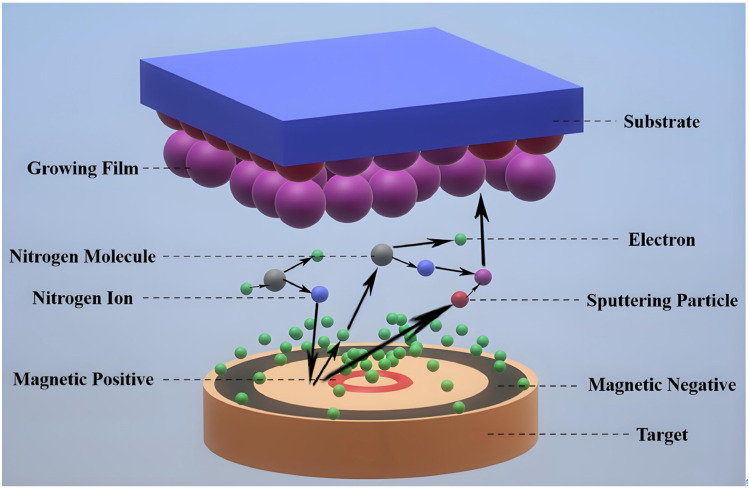
Schematic view of the MS experimental setup [105].

MS represents a substantial advantage as a pre-treatment process for Mg alloys, resulting in a relatively dense, uniform, and stable surface for the MAO process. This results in an improved coating quality and ensures an even distribution throughout the magnesium alloy surface, with minimal defects or weak areas. Moreover, this technology enhances the bonding force between the coating and the substrate, effectively extending the service life of the coating. Moreover, the composition and structure of the sputtered layer can be modified to enhance the performance of the MAO coating, thereby meeting diverse application requirements [87,88]. As illustrated in Figure 9, the fundamental principle of MS is that an applied potential difference within the deposition chamber causes argon ions (plasma) to undergo ionization, acceleration, and orientation towards the target. The plasma ions are capable of moving the target atom or molecule by either directly colliding with it or triggering a “collision cascade” that causes the atom to emit a particle, which then condenses into a coating on the substrate (anode). As demonstrated by Wei et al. [87], MS of a pure aluminum layer prior to MAO on AZ31 Mg alloy can result in the formation of coatings with varying morphologies and phase compositions. The coating prepared with aluminates as the MAO electrolyte is primarily composed of irregular nodules and the fine pores of α-Al_2_O_3_ and γ-Al_2_O_3_, lacking a pancake-like structure. In contrast, the coating prepared with silicates as the MAO electrolyte is characterized by a porous and pancake-like structure dominated by γ-Al_2_O_3_. Furthermore, compared to direct MAO on the AZ31 substrate, MAO carried out on a MS Al layer results in the formation of denser γ-Al_2_O_3_, leading to improved compactness. The oxidation process yields a more dense Al_2_O_3_ (PBR of 1.28 [104]), indicating that MS can enhance the corrosion resistance of MAO coatings. Hu et al. [88] demonstrated that the initial deposition of a dense pure aluminum layer approximately 11 μm thick via MS onto the surface of an AZ31 substrate, obtaining an oxide dominated by γ-Al_2_O_3_ through MAO, resulted in distinctly improved corrosion resistance for the MAO coating, as evidenced by an *I_corr_* value of only 3.93 × 10^−6^ A·cm^−2^ after 5 min, in comparison to 2.54 × 10^−4^ A·cm^−2^ for the substrate.

### 2.3. Cold Spray

**Figure 10:**
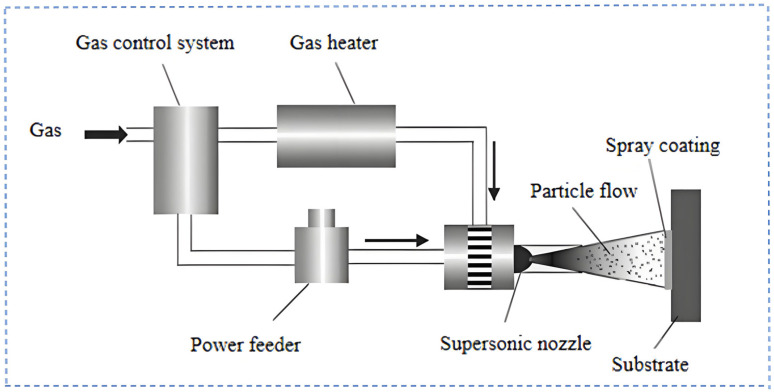
Principle diagram of CS process [108].

**Figure 11:**
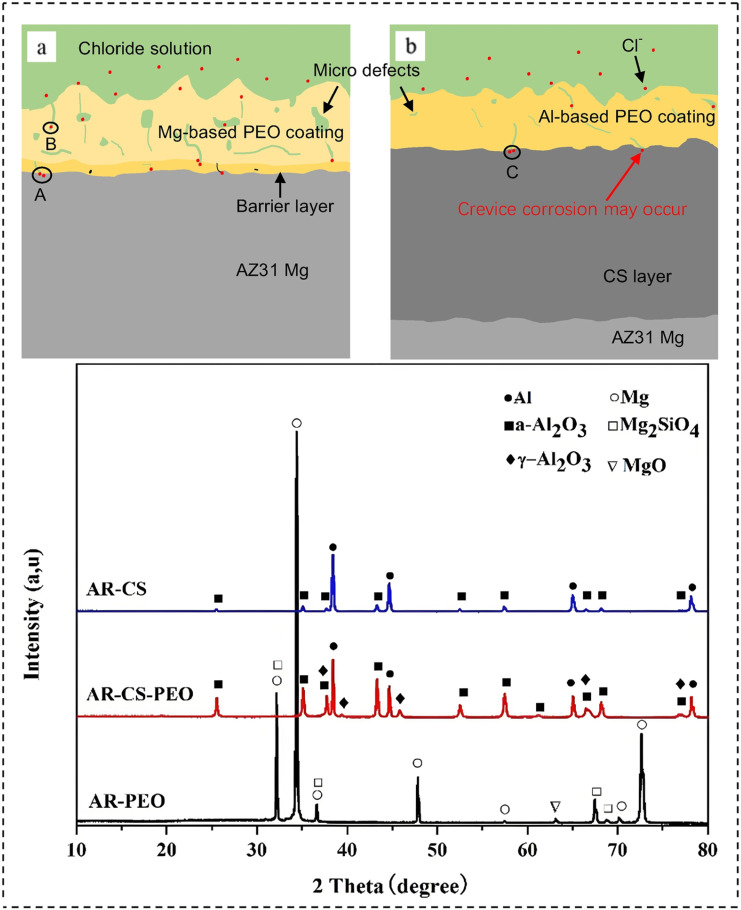
Cross-sectional schematics of the corrosion process in chloride solution: AR-MAO sample (**a**); AR-CS-MAO sample (**b**); The XRD patterns of CS and MAO coatings [89].

Figure 10 provides a visual representation of the operational mechanism of the CS technology. In the initial phase, a compression device applies pressure to the gas, which subsequently flows through a heater to increase its temperature. At the nozzle outlet, the gas undergoes a rapid expansion, reaching velocities that exceed the speed of sound. The high-pressure and heated gas at the front of the spray gun is used to accelerate metal particles to supersonic speeds, which ultimately results in their impact and adhesion to the surface of the substrate material. The CS method, which is powered by gas, achieves plastic deformation and particle deposition through the high-speed collision of micron-sized solid particles with the substrate. This process results in the solidification of particles, which form a coating through mechanical interlocking and local metallurgical bonding between the particles [106,107]. This enhances the corrosion resistance of the material. In the field of corrosion protection for magnesium alloys, CS has attracted considerable interest due to its remarkable resistance to corrosion. CS is capable of depositing a layer of metal or alloy with similar physicochemical properties as the substrate onto the surface of Mg alloys, including layers of copper and aluminum. The deposited layer not only integrates closely with the substrate but also plays a fundamental role in subsequent MAO processes by improving the bonding between the substrate and MAO coatings, thus enhancing adhesion and durability. Concurrently, the metal deposition layer may exhibit characteristics such as corrosion resistance and wear resistance, which are retained and further augmented during the MAO process, thereby markedly enhancing the overall performance of the Mg alloy. As illustrated in Figure 11, Rao et al. [89] fabricated a composite coating on AZ31 Mg alloy through the application of CS and MAO techniques. Notably, the coating in the CS pre-treated group exhibited greater density than that in the unpre-treated group, effectively obstructing the intrusion of corrosive media. Following CS pre-treatment, the *E_corr_* of MAO increased from −1.453 V to −1.153 V, indicating that CS pre-treatment substantially enhanced the corrosion resistance of the Mg alloy after MAO treatment. This not only corroborates the efficacy of CS technology in protecting Mg alloys against corrosion but also provides novel insights and strategies for developing high-performance protective coatings for Mg alloys.

### 2.4. Other Pre-Treatment Processes

**Figure 12:**
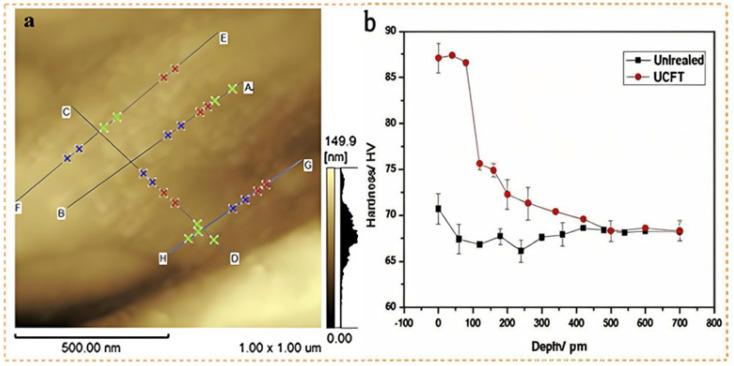
Grain size of the sample treated by UCFT, the largest size of the nanograin is on the C–D line, which is 73.47 nm while the average size of the nanograin is 48.67 nm (**a**); micro-hardness variation along the depth (**b**) [94].

In the context of pre-treatment processes, both SP and ultrasonic cold forging technology (UCFT) have been demonstrated to be effective in inducing plastic deformation of the substrate’s surface. This manipuanodizationation achieves grain refinement and establishes the foundation for a high-quality substrate that is conducive to MAO processes [90,109,110]. The findings of Daniel et al. [93] have demonstrated that Mg alloys treated with SP are capable of generating denser coatings during MAO. The micro-pits and imperfections introduced by SP serve as nucleation sites for the coating material, leading to a denser structure. Moreover, a meticulously controlled SP regimen enhances the substrate’s surface reactivity, resulting in a proliferation of sites with favorable thermodynamic properties. This manipulation ultimately enhances the overall performance and efficiency of the system, thereby creating propitious conditions for the formation of MAO coating nuclei. Compared to MAO without pre-treatment, SP followed by MAO resulted in a decrease in the *I_corr_* of the coating from 7.11 × 10^−7^ A·cm^−2^ to 2.14 × 10^−7^ A·cm^−2^. Chen et al. [94] demonstrated that UCFT pre-treatment combined with MAO distinctly enhanced the corrosion resistance of AZ31B Mg alloy. As depicted in Figure 12, the surface structure of the samples treated with UCFT and MAO was notably improved, and the grain size was refined to 30–80 nm. The corrosion resistance of the samples treated with a combination of UCFT and MAO was enhanced compared to those treated with single MAO due to a variety of phases present in the coatings, such as Mg_3_(PO_4_)_2_, tertiary calcium phosphate, CaHPO_4_·2H_2_O, HA, Mg, and MgAl_2_O_4_, among other compounds, which contributed to an enhanced chemical stability and corrosion resistance.

The method of chemical conversion coatings (CCC) plays a pivotal role as a preliminary step prior to the MAO of Mg alloys, achieving the effect of cleaning and activation through the reaction or adsorption of active ingredients in the solution with the surface of Mg alloys. This process may result in the formation of a chemical conversion coating on the substrate surface [95]. A study by Hariprasad et al. [95] presents substantial evidence that the chemical conversion coating method is an effective pre-treatment process for Mg alloy MAO. The *I_corr_* of the composite coating, formed by combining cerium conversion and MAO on the surface of AZ31 Mg alloy, is two orders of magnitude lower than that of a coating prepared by MAO alone. This notable enhancement can be primarily attributed to the high thickness and densification of the composite coatings. In conclusion, the use of chemical CCC as a pre-treatment process for MAO has been demonstrated to markedly enhance corrosion resistance in the resulting coatings.

### 2.5. Conclusion of This Part

Pre-treatment processes play a crucial role in the preparation of MAO coatings, as they have the ability to modify the microstructure of the alloy, enhance the bonding force between the coating and the substrate, and improving coating performance to meet the requirements of complex application environments. Therefore, when selecting pre-treatment processes, consideration should be given to alloy composition, application scenarios, performance requirements, and cost-effectiveness, in order to optimize coating performance.

## 3. Effect of Post-Treatment Processes

**Figure 13:**
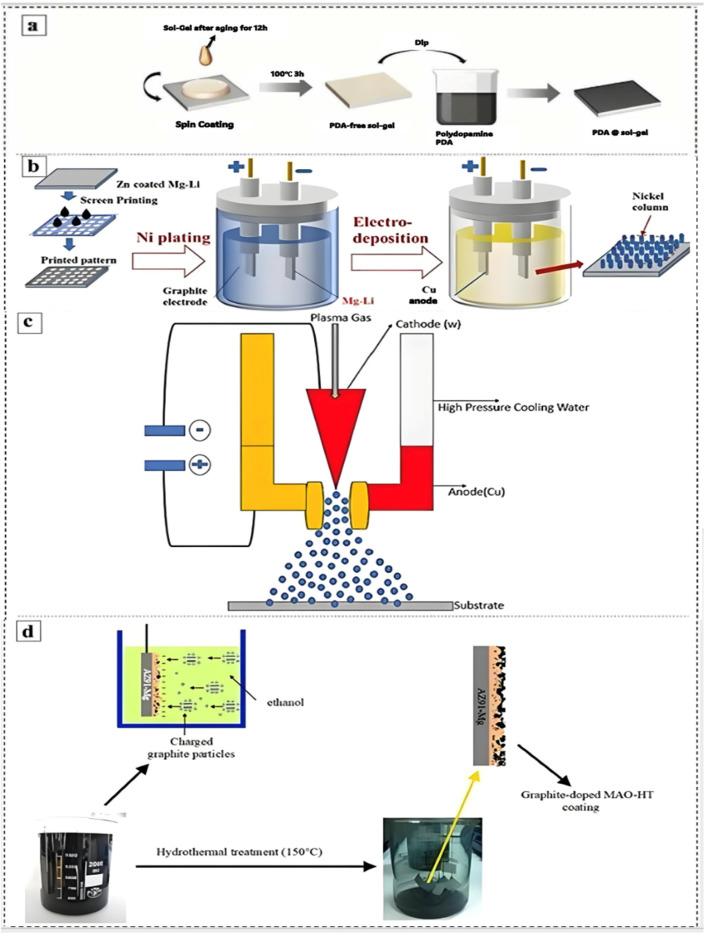
Schematic diagram of post-treatment operation for MAO of Mg alloy. Sol-gel (**a**) [133], EPD (**b**) [134], Air plasma spray (**c**) [135], HTT (**d**) [136].

Improving the corrosion resistance of MAO coatings on Mg alloys by post-treatment processes is one of the current research hotspots [111,112,113,114,115,116,117,118,119,120,121,122,123,124,125,126,127]. The most commonly employed post-treatment processes include impregnation [54], sol-gel [127], electrophoretic deposition (EPD) [54,128,129], and hydrothermal treatment (HTT) [130,131,132]. A schematic representation of the various post-treatment processes is provided in Figure 13, while the resulting coatings are described in detail in the following section. The impact of different post-treatment processes on the *E_corr_* and *I_corr_* of the MAO coatings on Mg alloys is summarized in Table 3. The results demonstrate that post-treatment can, to a certain extent, contribute to an improvement in the corrosion resistance of MAO coatings.

### 3.1. Impregnation

**Figure 14:**
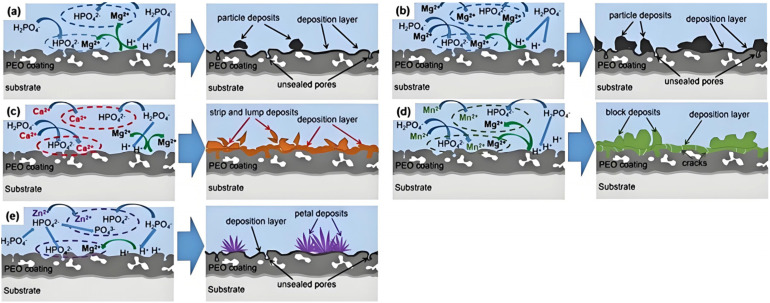
Schematic diagram of the sealing process for P-MAO (**a**); Mg-P-MAO (**b**); Ca-P-MAO (**c**); Mn-P-MAO (**d**); Zn-P-MAO (**e**) [111].

Hydroxides or other compounds could be formed on the surface of MAO coatings during the impregnation process. These compounds serve to fill the micro-pores and micro-cracks in the coatings, effectively preventing the penetration of corrosive media. Simultaneously, impregnation strengthens the adhesion between the coating and the substrate, resulting in an overall enhancement of coating performance [111,112,113,114,115,116,117]. Phosphate-based buffers are commonly utilized under acidic conditions and are frequently employed in impregnation due to their unique chemical properties. It has been observed that, while phosphate-based solutions may result in partial coating dissolution, precise control of the impregnation time can effectively manage the degree of coating dissolution and deposition, thereby achieving optimal coating performance [111,112]. This is evidenced by the work of Qian [111], who immersed Mg samples post-MAO in phosphate solutions containing various cations, leading to a notable reduction in micro-pore size on the coating surface and an enhancement in densification. Figure 14 visually depicts the corrosion mechanism of MAO coatings following impregnation with NaH_2_PO_4_ solution, wherein HPO_4_^2−^ from H_2_PO^4−^ ionization reacts with Mg^2+^ to form a deposit layer of MgHPO_4_, which effectively seals the pores of the coating and improves its corrosion resistance. Qian et al. [112] demonstrated that impregnation with a solution containing copper salts and phosphate salts substantially improved the corrosion resistance of MAO coatings on AZ91D Mg alloy. The presence of MgHPO_4_ crystalline phase in the coating after sealing treatment in a solution with added NaH_2_PO_4_ resulted in a considerable reduction in the depth of corrosion pores and the number of corrosion products in the severely corroded area, compared to unimpregnated MAO coatings, after 384 h of immersion in 3.5 ωt% NaCl. In a similar study, Qian et al. [113] conducted phosphate impregnation on AZ91D MAO coatings and observed that the surface became smoother and pore size decreased post-treatment. Furthermore, upon sealing for 15 min, the coating obtained a dense structure with MgHPO_4_ as its main composition. The *I_corr_* value for phosphate phosphate-impregnated MAO was approximately one order of magnitude lower than that for unimpregnated treatment.

Rare earth elements, renowned for their exceptional physicochemical properties, have the potential to enhance the overall performance of MAO coatings. Recently, researchers have concentrated their efforts on investigating the impact of incorporating rare earth elements into the sealing treatment on the corrosion resistance of MAO coatings. Mohedano et al. [114] observed the formation of a Ce-rich layer on the surface of MAO coatings on Mg-Y-Zn alloy substrates following treatment with Ce salts, which effectively reduced the number of coating pores. This phenomenon can be attributed to the oxidation of Ce^3+^ ions by H_2_O_2_ in the sealing solution (Equation (1)) and the partial dissolution of MgO and Mg_2_SiO_4_ in acid, which results in local alkalinity and the formation of insoluble CeO_2_ (Equations (2)–(4)). The enhanced corrosion resistance can be ascribed to the blockage of pores within the coating structure by the accumulation of CeO_2_.

(1) $$2{Ce}^{3+}\left(aq\right)+H_{2}O_{2}+2{OH}^{-}\left(aq\right)\to 2{{Ce\left(OH\right)}_{2}}^{2+}$$

(2) $$MgO\left(s\right)+2H^{+}\left(aq\right)\to {Mg}^{2+}\left(aq\right)+H_{2}O\left(l\right)$$

(3) $${Mg}_{2}{SiO}_{4}\left(s\right)+4H^{+}\left(aq\right)\to 2{Mg}^{2+}\left(aq\right)+{SiO}_{2}\left(aq\right)+2H_{2}O\left(l\right)$$

(4) $${{Ce\left(OH\right)}_{2}}^{2+}\left(aq\right)+2{OH}^{-}\left(aq\right)\to {Ce\left(OH\right)}_{4}\left(s\right)\to {CeO}_{2}\left(s\right)+2H_{2}O\left(l\right)$$

Mingo et al. [116] conducted a study that further validated the beneficial impact of Ce-based sealing on the corrosion resistance of MAO coatings. The researchers observed that Ce salt treatment induced a dissolution/precipitation reaction on the MAO surface, leading to the formation of Ce-rich compounds (CeO_2_). These compounds effectively obstructed the ingress of corrosive media into the coating, thereby augmenting its corrosion resistance. Additionally, Sun et al. [117] demonstrated a huge reduction in *I_corr_* for MAO-Ce(NO3)3 coatings treated with impregnation, compared to unimpregnated MAO coatings, when exposed to 3.5 ωt% NaCl solution. This improvement can be primarily attributed to the insoluble cerium oxide formed during the MAO process, which efficiently sealed micro-pores in the MAO coating and prevented penetration of corrosive solutions into the substrate region, consequently enhancing its corrosion resistance. Phuong et al. [118] investigated the impact of sealing AZ31 Mg alloys coated with MAO coatings using Ce and phosphate solutions. The results of the study demonstrated that the *I_corr_* of the samples that had undergone a sealing treatment with cerium salt decreased by one order of magnitude in comparison to the *I_corr_* of MAO, which was 125.9 × 10^−8^ A·cm^−2^. This led to an apparent enhancement in corrosion resistance. Pezzato et al. [115] evaluated the impact of Nd salt sealing post-treatment on the corrosion resistance of AZ91-coated Mg alloy samples through electrochemical testing. The findings indicated that the Nd salt sealing treatment notably enhanced the corrosion resistance of the coatings, primarily due to the effective physical barrier provided by the sealing layer, which effectively impeded the penetration of corrosive substances.

**Figure 15:**
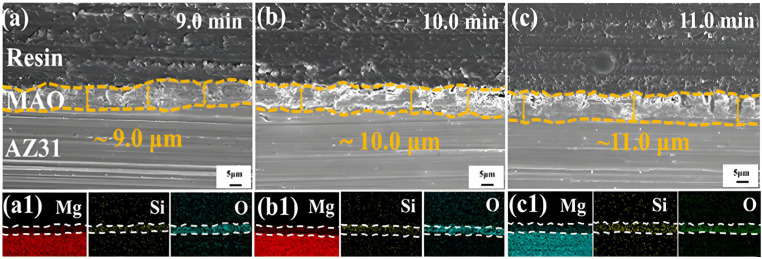
(**a**–**c**) Cross-sectional SEM Images & (**a1**–**c1**) Elemental Maps of MAO-(SA + Ce) Composite [119].

The preference for organic coatings among researchers is based on their exceptional chemical resistance and advanced protective properties. These coatings have become a prevalent post-treatment process for enhancing the corrosion resistance of Mg and its alloy MAO coatings. In comparison to traditional inorganic electrolytes, organic electrolytes are environmentally friendly and meet the criteria of the requirements of clean production [119,120,121,122]. Liu et al. [119] successfully fabricated MAO-(SA + Ce) coatings for AZ31 Mg alloys by impregnating them with stearic acid, hydrogen peroxide, and cerium nitrate dissolved in ethanol. The self-healing mechanism of MAO-(SA + Ce) composite coatings is characterized in Figure 15, depicting cross-sectional morphology consisting of a MAO coating (≈4.0 μm) and a newly formed Ce conversion coating (≈2.5 μm) on the exterior. Additionally, the *I_corr_* of the MAO-(SA + Ce) composite coating was reduced by 3–4 orders of magnitude compared to direct MAO coating. Dou et al. [120] conducted a study on the impact of chitosan on the corrosion resistance of MAO coatings, and observed that the sealing coating effectively closed the micro-pores of the MAO coatings, thereby enhancing their corrosion resistance. Štrbák et al. [121], on the other hand, investigated the effect of aqueous-based preservative containing corrosion inhibitors on the corrosion behavior of MAO coatings and found that the sealing treatment was capable of retaining water-based corrosion inhibitor-containing preservatives in MAO porous coatings, thus effectively preventing corrosion in aqueous solutions with varying aggressive chloride ions. Pak et al. [122] utilized phytic acid/3−aminopropyltrimethoxysilane (APTES) as an effective corrosion inhibitor to prevent the corrosion of MAO coatings. Through APTES hybridization for surface treatment of Mg alloys, composite coatings were obtained exhibiting excellent corrosion resistance with an *E_corr_* value of −1.566 V in 3.5% NaCl solution. Gnedenkov et al. [123] treated MA alloys using 8-hydroxyquinoline (8-HQ) solution to modify the MAO coating, resulting in overtly enhanced protective properties of the coating. The corrosion current value of the treated coating was 86 nA·cm^−2^, which is one order of magnitude lower than that of the MAO coating (810 nA·cm^−2^), indicating an enhanced corrosion resistance after post-treatment. Xue et al. [124] developed a MAO/CIP−PMTMS composite coating, leveraging the hydrophobicity and barrier effect of organics to achieve an *I_corr_* of 7.13 *×* 10^−8^ A·cm^−2^, distinctly lower than that of the MAO coating (3.54 *×* 10^−7^ A·cm^−2^). Toorani et al. [125] further improved the corrosion resistance of MAO coatings by treating them with a three-layer composite coating of silane and epoxy, resulting in coatings with higher densification and thickness.

### 3.2. Sol-Gel

The sol-gel technique, when used as a post-treatment process, effectively seals the holes in the MAO coating of Mg alloys, offering advantages such as strong coating adhesion, good barrier effect, and environmental protection [126]. For instance, Li et al. [127] demonstrated the preparation of HDTMS/SiO_2_ composite coatings on AZ91 Mg alloy surfaces pre-treated with MAO through hydrophobicity and sol-gel processes. The study revealed that the interface between the MAO coating and the gel hydrophobic layer was not distinct, indicating that the overall coating treated by the sol-gel process was relatively dense and distinctly improved the corrosion resistance of the samples. This improvement can be attributed to two possible reasons: First, by replacing hydrogen in hydroxyl groups with Si-(CH_2_)_15_CH_3_, the capillary pressure within silica networks is less negative, transforming Si-OH into Si-O chains, effectively preventing gel coating cracking and reducing substrate corrosion tendency; second, grafting a long carbon chain (−(CH_2_)_15_CH_3_) onto silica forms a hydrophobic layer, enhancing the ability of the sol-gel layer to inhibit corrosive ion penetration. Furthermore, Li et al. [140] utilized a combination of MAO technology and the sol-gel method to enhance corrosion resistance in Mg−lithium alloys. The findings indicated that the composite coatings, prepared using MAO technology and the sol-gel method, exhibited favorable densification, minimal defects, and obviously enhanced corrosion resistance in Mg alloys. Malayoglu et al. [141] employed sol-gel treatment as a post-treatment process to develop composite coatings for Mg alloys with micro-oxidation coatings, aiming to improve their corrosion resistance. The results demonstrated that the sol-gel process effectively sealed the MAO coatings, resulting in enhanced corrosion resistance.

### 3.3. HTT

HTT, which is recognized as an environmentally sustainable and cost-effective method, has attracted remarkable attention from both domestic and international researchers and scholars [130,142]. Malekkhouyan et al. [142] conducted the synthesis of LDH coatings on AZ31 Mg alloy based on MAO coatings through HTT. Their salt spray experiments revealed that no substantial changes were observed on the sample surfaces after 7 days of MAO treatment followed by HTT, while the coating of a single MAO sample had been completely deteriorated. Consequently, it can be concluded that HTT effectively enhances the corrosion resistance of Mg alloy MAO coatings. Zhang et al. [88] employed HTT for the post-treatment of Mg alloy MAO samples. The findings revealed a six-order increase in the R_CT_ value (a measure of corrosion resistance) of the hydrothermally treated Mg alloy MAO coating compared to that of the direct MAO coating without HTT. Dai et al. [131] successfully prepared a new MgAlY Layered Double Hydroxides-LDHs coating containing salicylate on the surface of AZ31 alloy MAO coating by using one-step hydrothermal method and intercalation method. The reaction process for in situ growth of MgAlY−LDHs/salicylate coating on AZ31 alloy was analyzed as depicted in Equations (5)–(11):

(5) $$2Mg\left(s\right)+O_{2}\to 2MgO\left(s\right)$$

(6) $$3{Mg}^{2+}\left(aq\right)+2{PO}_{4}^{3-}\left(aq\right)+22H_{2}O\left(aq\right)\to {{Mg}_{3}\left({PO}_{4}\right)}_{2}•22H_{2}O\left(s\right)$$

(7) $$4Al\left(s\right)+3O_{2}\left(g\right)\to 2{Al}_{2}O_{3}\left(s\right)$$

(8) $$MgO\left(s\right)+H_{2}O\left(aq\right)\to {Mg\left(OH\right)}_{2}\left(s\right)↓$$

(9) $${Al}_{2}O_{3}\left(s\right)+3H_{2}O\left(aq\right)+2{OH}^{-}\left(aq\right)\to 2{{Al\left(OH\right)}_{4}}^{-}\left(aq\right)$$

(10) $$Mg\left(s\right)+2Al\left(s\right)+8H_{2}O\left(aq\right)\to {Mg\left(OH\right)}_{2}\left(s\right)↓+2{Al\left(OH\right)}_{3}\left(s\right)↓+4H_{2}↑$$

(11) $${Al\left(OH\right)}_{3}\left(s\right)+{Al}^{3+}+{OH}^{-}\left(aq\right)\to {2Al{\left(OH\right)}_{4}}^{-}\left(aq\right)$$

The MLYS specimen (salicylic acid embedded + HTT + MAO) exhibited an overtly high coating resistance value of 1.83  *×* 10^6^ Ω·cm^2^. The MgAlY−LDHs/salicylic acid films grown on MAO and post-treated Mg alloys demonstrated excellent corrosion resistance, attributed to the enhanced densification and self-healing properties of the coatings, thereby ensuring the corrosion protection of the Mg alloys. Wang et al. [132] employed a hydrothermal growth method for in situ formation of MgAl−LDHs coating on the surface of MAO-coated Mg alloy. The composite coating of MAO−HTT demonstrated superior short-term and long-term protective properties compared to the MAO coating. The R_ct_ value of the composite coating is less negative one order of magnitude in comparison to that of the MAO coating, which can be attributed to the sealing effect of LDHs on the micro-pores of the MAO coating. Consequently, HTT has been shown to enhance the corrosion resistance of Mg alloy MAO coatings. In addition to sealing micro-pores, HTT exhibits ion exchange properties between LDH layers, enabling attraction or adsorption of corrosive ions such as Cl^−^, thereby further mitigating their corrosive impact on Mg alloy substrates.

### 3.4. Spraying and Electrodeposition

The coating method employs a range of techniques, including electrodeposition, Air Plasma Spray (APS), and others, to create a protective coating on metal or other materials. This process is primarily employed to enhance the corrosion resistance, wear resistance, electrical conductivity, and other properties of the base materials. Farshid et al. [139] employed MAO and electrodeposition to fabricate MAO/PDA composite coatings on AZ91 Mg alloy. These coatings exhibited self-healing capabilities, enhanced the corrosion resistance and bioactivity of the alloy. A uniform, rough, dense, and hydrophilic MAO−PDA coating was electrodeposited at a dopamine concentration of 1 mg/mL (120 T−1C) with an *I_corr_* of 4.31 *×* 10^−10^ A·cm^−2^, which was obviously lower than that of the MAO sample (3.53 *×* 10^−8^ A·cm^−2^). Nadaraia et al. [143] combined MAO and fluorinated polymer spraying to prepare coatings on Mg−Mn−Ce alloys. The application of super-dispersed polytetrafluoroethylene (SPTFE) resulted in an enormous reduction in coating porosity from 18% to 2% after three applications. The incorporation of fluorine into the MAO layer validates the effectiveness of the fluoropolymer in sealing micro-defects within the MAO coating, indicating that the composite method can greatly enhance the corrosion resistance of the alloy [144]. Furthermore, Mohammadreza et al. [144] employed APS as a post-treatment process following MAO, with the objective of enhancing the corrosion resistance and antimicrobial activity of Mg alloys. The APS-treated coating is comprised of an inner barrier layer, an outer porous layer, and a nano-structured zirconium dioxide layer. Furthermore, it was observed that molten zirconium dioxide particles infiltrated into the outer porous layer of the MAO coating, effectively expelling air present in micro-pores and thereby sealing the MAO coating, thus improving its corrosion resistance.

### 3.5. Other Post-Treatment Process

As a post-treatment method, laser surface processing can effectively address the surface treatment challenges of various materials. Wang et al. [119] demonstrated that laser processing can markedly reduce the number and size of pores on the surface of MAO coatings, thereby enhancing the corrosion resistance of the coatings. EPD is an electrochemical method used to deposit charged particles onto the electrode surface to form a coating [145]. The porous structure of MAO coatings necessitates additional polymer deposition at the bottom of the pores, and electrophoresis embeds particles into the pores to create a robust composite coating. Studies have shown that EPD of ZnO nanoparticles [54], Polytetrafluoroethylene (PTFE) [128], and Graphene oxide (GO) [129] in MAO coatings has resulted in pore sealing effects. The findings indicated that ZnO nanoparticles can be embedded in coating pores to form a dual-layered coating through physical interlocking, thereby improving corrosion resistance [54]. PTFE was utilized to seal the pores, thereby increasing the coating thickness and barrier property to enhance corrosion resistance [128]. GO effectively seals MAO micro-pores and micro-cracks, leading to a more dense coating and enhanced corrosion resistance [129]. MS, a widely employed method of physical vapor deposition (PVD), offers numerous advantages including high coating purity, efficient deposition, uniformity of deposited coating, and uniform and effective coverage of the sample [146].

### 3.6. Conclusions

The application of suitable post-treatment processes can effectively fill the surface micro-pores and micro-cracks of MAO coatings, thereby enhancing their densification and corrosion resistance. The sol-gel technique has been demonstrated to markedly enhance both the densification and bonding strength of the coating, resulting in augmented long-term corrosion resistance. The HTT and plating methods are effective in improving the corrosion resistance of MAO coatings because they provide superior sealing and can achieve substantial coating thickness. When selecting appropriate methods, it is essential to consider these factors along with the intended application, in order to optimize both the corrosion resistance and overall performance of the coating.

## 4. Summary and Prospect

In conclusion, the combination of MAO with pre- or post-treatment process can markedly enhance the corrosion resistance of Mg alloys in a variety of corrosive environments. Firstly, pre-treatment (laser treatment, SP, and UCFT) optimizes the microstructure of the Mg alloy surface and improves coating adhesion and densification, thereby enhancing corrosion resistance. Secondly, pre-treatment (MS and CS) entails the deposition of a coating layer on Mg alloy, which modifies the composition of the MAO coating layer and forms a denser substance, thereby enhancing corrosion resistance. Thirdly, post-treatment (impregnation, sol-gel, EPD, MS and HTT) serve to seal micro-pores and micro-cracks in the MAO coating, thereby regulating its structure and improving corrosion resistance. Furthermore, specific post-treatment processes result in the formation of a more compact layer on the MAO coating surface, thereby creating an additional barrier against corrosion.

It is anticipated that this technique will be adopted more widely in industries where corrosion resistance is a critical requirement as the field of research progresses. Nevertheless, further investigation and optimization are required to ascertain the long-term efficacy of Mg alloy MAO and the combined application of pre-treatment process and post-treatment coatings. This can be achieved by optimizing the utilization of the composite treatment method through the strategic timing and methods of pre-treatment, as well as the agents, techniques, and duration of post-treatment processes. Presently, post-treatment process agents are primarily inorganic compounds. However, given the complex nature and varied performance of organic matter, further comprehensive investigation is required to ascertain its potential use as a post-treatment agent for Mg alloys. There is a paucity of research examining the impact of factors such as temperature and stress on long-term corrosion resistance. It is of the utmost importance to address these issues, in order to establish a basis for industrial use of the experimental process. It is also of the utmost importance to select post-treatment agents that are environmentally friendly and meet the requirements of the experimental process. The development of environmentally friendly post-treatment processes or the utilization of natural compounds will not only contribute to the sustainable preparation of coatings but will also have a remarkable positive impact on the industrialization process.

## Figures and Tables

**Table 3:** *E_corr_* & *I_corr_* of MAO Coatings on Mg Alloys with/without Post-treatment.

Substrate	Composition of the MAO Coatings	MAO Coatings Without Post-Treatment Process		Post-Treatment Process	MAO Coatings with Post-Treatment Process		Ref.
		*E_corr_* (V)	*I_corr_* (A·cm^−2^)		*E_corr_* (V)	*I_corr_* (A·cm^−2^)	
Mg	Mg, MgO, Mg_2_SiO_4_	1.349	7.72 × 10^−7^	Impregnation	−1.29	5.87 × 10^−8^	[111]
AZ31	Mg_3_(PO_4_)_2_, MgO, Mg				−0.69	6.72 × 10^−6^	[137]
AZ31	Mg, MgO		2.47 × 10^−6^			9.28 × 10^−8^	[138]
AZ91D	Mg, Mg_2_SiO_4_, MgO	−1.32	5.72 × 10^−7^		−1.31	3.34 × 10^−8^	[113]
LPSO Mg-Y-Zn	Mg, MgO	−1.61	7.1 × 10^−8^		−1.2	2 × 10^−9^	[114]
cp-Mg	Mg, MgO, Mg_2_SiO_4_	−1.59	1.4 × 10^−5^		−1.61	3.7 × 10^−6^	[117]
AZ31		−1.38	1.259 × 10^−6^		−1.36	1.34 × 10^−7^	[118]
AZ31B		−1.44	3.9 × 10^−7^		−1.32	1.81 × 10^−8^	[122]
MA8		−1.51	8.1 × 10^−7^		−1.44	8.6 × 10^−8^	[123]
AZ31		−0.66	2.33 × 10^−6^		−0.59	8.95 × 10^−10^	[123]
AZ31B	MgO, Mg_3_(PO_4_)_2_, Mg(OH)_2_, Al_12_Mg_17_, Mg	−1.60	3.53 × 10^−8^	HTT	−1.35	1.27 × 10^−8^	[139]
AZ31	Mg_3_(PO_4_)_2_, Mg, MgO					2.98 × 10^−10^	[131]
AZ31	MgAl_2_O_4_, Mg	−1.29	7.62 × 10^−8^		−1.33	1.97 × 10−9	[132]
Mg alloy	Mg_3_(PO_4_)_2_, Mg, MgO	−1.75	6.17 × 10^−6^	EPD	−1.42	4 × 10^−8^	[54]
AZ91	Mg, MgO, Mg_3_(PO_4_)_2_	−1.54	9.86 × 10^−6^		−0.87	1.75 × 10^−7^	[129]
AZ31	Mg, MgO	−1.33	1.54 × 10^−6^	Sol− gel	−1.09	1.05 × 10^−7^	[127]
Mg−Li		−1.38	5.64 × 10^−7^		−1.38	5.64 × 10^−7^	[140]
AM50B	Periclase (MgO), Spinel MgAl_2_O_4_, Mg				−1.39	6.57 × 10^−9^	[141]

Note. HTT−Hydrothermal Treatment; EPD−Electrophoretic Deposition.

## Data Availability

No new data were created or analyzed in this study. Data sharing is not applicable to this article.
